# Vitamin D Insufficiency and Bone Mineral Status in a Population of Newcomer Children in Canada

**DOI:** 10.3390/nu5051561

**Published:** 2013-05-14

**Authors:** Hassanali Vatanparast, Christine Nisbet, Brian Gushulak

**Affiliations:** 1 Division of Nutrition and Dietetics, College of Pharmacy and Nutrition, University of Saskatchewan, Saskatoon, SK S7N 5A2, Canada; 2 School of Public Health, University of Saskatchewan, Saskatoon, SK S7N 5A2, Canada; 3 Division of Nutrition and Dietetics, College of Pharmacy and Nutrition, University of Saskatchewan, Saskatoon, SK S7N 5A2, Canada; E-Mail: christine.nisbet@usask.ca; 4 Migration Health Consultants, Qualicum Beach, British Columbia, V9K 1S9, Canada; E-Mail: brian.gushulak@gmail.com

**Keywords:** vitamin D, newcomer immigrant children, bone mineral status

## Abstract

Background: Low levels of circulating vitamin D are more likely to be found in those with darker skin pigmentation, who live in areas of high latitude, and who wear more clothing. We examined the prevalence of vitamin D deficiency and inadequacy in newcomer immigrant and refugee children. Methods: We evaluated circulating vitamin D status of immigrant children at the national level. Subsequently, we investigated vitamin D intake, circulating vitamin D status, and total body bone mineral content (TBBMC) in newcomer children living in Saskatchewan. Results: In the sample of newcomer children in Saskatchewan, the prevalence of inadequacy in calcium and vitamin D intakes was 76% and 89.4%, respectively. Vitamin D intake from food/supplement was significantly higher in immigrants compared to refugees, which accords with the significant difference in serum status. Circulating vitamin D status indicated that 29% of participants were deficient and another 44% had inadequate levels of serum 25(OH)D for bone health. Dietary vitamin D intake, sex, region of origin, and length of stay in Canada were significant predictors of serum vitamin D status. Results for TBBMC revealed that 38.6% were found to have low TBBMC compared to estimated values for age, sex, and ethnicity. In the regression model, after controlling for possible confounders, children who were taller and had greater circulating vitamin D also had greater TBBMC. Nationally, immigrant children, particularly girls, have significantly lower plasma 25(OH)D than non-immigrant children. Interpretation: Newcomer immigrant and refugee children are at a high risk of vitamin D deficiency and inadequacy, which may have serious negative consequences for their health.

## 1. Introduction

Recent studies report various health benefits for vitamin D along with its important role in bone health [1]. Limited food sources of vitamin D and long winters resulting in reduced sun exposure in Canada have raised concerns, particularly in growing children where vitamin D is needed for bone mineral accrual. National data report a low prevalence of vitamin D deficiency in children aged 6–11 years compared to other age groups [2]. However, the growing proportion of immigrants in Canada, particularly children, may represent communities at greater risk for vitamin D deficiency. Some new arrivals have high skin pigmentation, and social and cultural factors that can result in insufficient vitamin D intake from food [3,4,5,6]. From 2002 to 2004, 150 cases of rickets were reported in Canadian children [7]. Darker-skinned individuals represented 89% of cases, while 24% were immigrants [7]. In a cross-sectional study in Edmonton, vitamin D concentrations decreased in children as age increased [8]. The authors speculated that this is possibly due to less consumption of milk fortified with vitamin D and less exposure to the sun [8]. In response to the paucity of information in this area, we evaluated circulating vitamin D status of immigrant and refugee children new to Canada. Additionally, we examined the association between circulating vitamin D with bone mineral status. 

## 2. Methods

Two studies were conducted to address the following research objectives: (i) to obtain a national perspective on vitamin D status of immigrant children in comparison to non-immigrant children; (ii) to evaluate determinants of vitamin D and its association with bone mineral status in children new to Canada.

### 2.1. Study 1. Vitamin D Status of Canadian Immigrant and Non-Immigrant Children

We used data from the Canadian Health Measures Survey (CHMS), cycle 1, 2007–2009, conducted by Statistics Canada [9]. The CHMS is a nationally representative survey among approximately 5500 Canadians aged 6–79 years, representative of 96.3% of Canadians. Circulating 25-hydroxyvitamin D [25(OH)D] levels were obtained for Canadian non-immigrant and first-generation immigrant children aged 6–11 years. In CHMS data, immigrants are not differentiated from refugees. The information on vitamin D measurement in the CHMS is found elsewhere [2]. Descriptive data are presented as mean ± SEM. A statistically significant difference in vitamin D status among immigrant *versus* non-immigrant children is indicated by no overlap in 95% Confidence Intervals [10]. As per Statistics Canada’s recommendation, analyses were weighted and bootstrapped to obtain estimates representative of the Canadian population. Degree of freedom was considered 11 due to sampling structure, and alpha was set at 0.05. Data manipulation, cleaning, and creating new variables were done using SPSS 19. All analyses were conducted by STATA SE 11. 

### 2.2. Study 2. Healthy Immigrant Children (HIC)

#### 2.2.1. Study Design and Participants

In the absence of official data sources that identifies immigration status in Saskatchewan, a convenience sample of study participants (*n* = 72) were recruited with the assistance of several organizations that have regular contact with immigrant and refugees. These organizations include Saskatoon Open Door Society, Saskatchewan Intercultural Association, and ethno-cultural associations. Participant recruitment posters were also placed in accessible public locations including the universities, public libraries, health care facilities and commercial locations. Interpreters were used for 50 (69%) participants while 22 (31%) were able to speak English and, therefore, did not require an interpreter. 

In a cross-sectional design (2010–2011), health and nutrition measures were collected from immigrant (*n* = 33) and refugee (*n* = 39) children aged 7–11 years who had been living in Saskatoon, Saskatchewan, Canada for no more than five years. An immigrant is someone who “comes as a permanent resident to a country other than one’s native land” [11]. A refugee is someone who “owing to well-founded fear of being persecuted for reasons of race, religion, nationality, membership of a particular social group or political opinion”, is unable or unwilling to return to his/her country of birth due to fear for their safety [12]. This age range is an important time for bone acquisition and for establishing healthy dietary habits. Ethics approval was obtained from the University of Saskatchewan Biomedical Research Ethics Board (Bio # 09-197).

#### 2.2.2. Data Collection

Data collection for the study included multiple variables. Demographics and socioeconomic status were evaluated using questionnaires from the Canadian Community Health Survey [13]. Food security status was evaluated using the version of the United States Department of Agriculture questionnaire that was modified by Canada [14].

Anthropometric measurements included height, weight and waist circumference. Values were recorded in centimeters to the nearest millimeter. All measurements were taken twice for consistency to help eliminate human error. Percentile Body Mass Index (BMI) was categorized as normal, overweight, or obese according to World Health Organization (WHO) child classifications [15]. 

To assess physical activity, we used the CHMS children’s physical activity questionnaire (CPA). Another variable was derived to examine sedentary activity in hours per day. Based on the CHMS, two questions on sun exposure were also included [9].

Three 24-h dietary recalls were administered to each child to obtain a usual intake. All 24-h recalls were administered at least three weeks apart and conducted in person. The child’s caregiver assisted in the dietary assessments. Diet analyses were performed in “The Food Processor Nutrition and Fitness Software version SQL 10.5”, Esha Research, Salem, USA. Foods were categorized into food groups consistent with *Eating Well with Canada’s Food Guide* [16]. To examine the overall nutrition status, we used the Canadian version of the Healthy Eating Index (HEIC) [17]. The HEIC has been validated and incorporates recommendations from *Eating Well with Canada’s Food Guide* [17]. This index categorizes diets into one of three categories, “poor”, “needs improvement”, and “good” [17]. 

#### 2.2.3. Outcome Measures

Blood samples were taken from 1 May to 30 September 2010 using a finger prick on eight 6-mm spots on a filter card. Circulating 25(OH)D was analyzed from dried blood spots, using a standard LC-MS/MS assay by ZRT Laboratory which is widely used for measuring 25-Hydroxy Vitamin D2/D3 [18,19]. The ZRT Laboratory participates in DEQAS, the Vitamin D Quality Assessment Scheme, which provides control samples to ensure assay accuracy [18,19]. Plasma and blood spot determinations were deemed to be equivalent because of the high stability of 25(OH)D in serum or plasma [20]. The blood spot card measurements of vitamin D levels are in agreement with serum and whole blood specimen [21]. Recent recommendations by the Institute of Medicine’s dietary reference intake (DRI) panel on vitamin D were used to define vitamin D status including less than 30 nmol/L as deficient, 30–50 nmol/L as inadequate, and more than 50 nmol/L as sufficient [22]. 

Bone mineral status was assessed using Dual-energy X-ray Absorptiometry (DXA), Hologic Inc., Discovery-Wi, Bedford, USA, Serial #80964. This study focused on Total Body Bone Mineral Content (TBBMC) as the most accurate measure in children [23]. The coefficient of variation for TBBMC in our laboratory is 0.5%. Values obtained for TBBMC were compared to estimated normal values for each child’s age, sex, and ethnicity based on data from four longitudinal studies [24]. 

#### 2.2.4. Statistical Analyses

Descriptive statistics were analyzed by calculating means and standard deviations of variables of interest as well as the distribution of participants in various categories. We used a two-sided independent Student’s *t*-test or non-parametric equivalent (Mann Whitney *U*-test) to investigate differences between refugees and immigrants and between males and females. Pearson’s Chi square was used to analyze categorical variables. Finally, multivariate analyses (linear and logistic regression) were conducted to examine the association between variables of interest and health outcomes (vitamin D status, TBBMC), controlling for possible confounders. Analyses were conducted using PASW Statistics 18 by Polar Engineering and Consulting, Chicago, USA. In all analyses, alpha was set at 0.05. All data, when possible, was compared to Canadian published data. 

## 3. Results

Descriptive data from CHMS revealed that mean plasma 25(OH)D concentrations in non-immigrant children was significantly higher than in immigrant children. This difference is over 20 nmol/L in immigrant girls (Table 1). 

**Table 1:** Plasma 25-hydroxyvitamin D [25(OH)D] (nmol/L) concentrations in immigrant and non-immigrant children aged 6–11 years in Canada ^1^.

Children aged 6–11 years	Plasma 25-hydroxyvitamin D [25(OH)D] (Mean ± SEM)
All children ( *n* = 1,817,260)	75.1 ± 2.4
Boys	79.9 ± 2.0
Girls	73.1 ± 3.0
Immigrant children ( *n* = 134,833)	61.1 ± 5.9 *
Immigrant boys	69.0 ± 7.9
Immigrant girls	54.1 ± 4.5 **
Non-immigrant children ( *n* = 1,682,427)	76.2 ± 2.2
Non-immigrant boys	77.5 ± 1.9
Non-immigrant girls	74.7 ± 2.8

^1^ Data from the Canadian Health Measures Survey Cycle 1, 2007–2009. * Significantly lower than non-immigrant children. ** Significantly lower than non-immigrant boys and girls.

In the HIC study, the mean ± SD age of participants was 8.9 ± 1.4 years, with no significant difference between immigrants (*n* = 33) and refugees (*n* = 39). There was also no significant difference in distribution of males (*n* = 48) and females (*n* = 24) according to immigration status. Most immigrant children in the study were from the Middle East (73%), while refugee children were from various regions including South East Asia, Africa, the Middle East and Latin America in descending order. In 2009, approximately 78% of newcomers in Saskatchewan were from Asia and the Pacific (59.9%), Africa and the Middle East (15.4%) and South and Central America (2.5%) [25]. These regions contribute to around 80% of immigrants in Canada [26]. The mean length of stay in Canada was 2.6 ± 1.5 years. Over 55% of families were from the lowest income bracket according to Statistics Canada classification. 

Table 2 presents general characteristics of participants. Percentile height (mean ± SD) was lower than the 50th percentile with no significant difference between immigrants and refugees. The majority of children were found to have a normal BMI while 22.2% were overweight and 6.9% were obese. Results showed 87.5% of immigrant and refugee newcomer children were obtaining the recommended number of hours per day of physical activity (≥60 min) according to the Canadian Society for Exercise Physiology [27]. However, many participants were spending too much time (>2 h/day) in sedentary activities such as time spent watching television or movies, playing video games, or using the computer.

**Table 2:** General characteristics of healthy immigrant children study participants.

Characteristics	Immigrants *n* = 33 (45.8%)	Refugees *n* = 39 (54.2%)	All participants *n* = 72 (100%)
Age (Mean ± SD)	8.9 ± 1.6	8.9 ± 1.3	8.9 ± 1.4
Sex			
Male	22 (66.7%)	26 (66.7%)	48 (66.7%)
Female	11 (33.3%)	13 (33.3%)	24 (33.3%)
Length of stay in Canada in years (Mean ± SD)	2.6 ± 1.5	2.5 ± 1.1	2.5 ± 1.3
Height in cm (Mean ± SD)	132.6 ± 13.2	130.3 ± 10.9	131.4 ± 12.0
Percentile Height (Mean ± SD)	49.7 ± 31.1	42.6 ± 30.8	45.8 ± 31.0
Weight in kg (Mean ± SD)	32.1 ± 10.6	29.9 ± 7.8	30.9 ± 9.2
Percentile Weight (Mean ± SD)	68.8 ± 27.4	64.9 ± 28.8	66.7 ± 28.0
Physical activity in h/week (Mean ± SD)	13.1 ± 5.4	12.0 ± 3.7	12.5 ± 4.6
Recommended level (≥60 min/day)	29 (87.9%)	34 (87.2%)	63 (87.5%)
Less than recommended level (<60 min/day)	4 (12.1%)	5 (12.8%)	9 (12.5%)

Dietary vitamin D and calcium intakes and HEIC are presented in Table 3. Only 24.2% of participants met recommendations for servings per day of milk and alternatives. The prevalence of calcium intake inadequacy was 76%. Vitamin D intake from food and supplement was 213 ± 195 IU (mean ± SD) for all participants with a prevalence of inadequacy of 89.4%. Only two children reported taking a vitamin D supplement regularly. Vitamin D intake from food and supplement was significantly higher in immigrants compared to refugees, which accords with the significant difference in serum status (Table 3). There was a significant difference in mean ± SD HEIC scores between immigrants and refugees at 65.4 ± 7.7 *vs.* 60.4 ± 8.8, respectively (*p* = 0.021). The majority of participants (90.9%) needed to improve their diet. Only one immigrant was classified in “good” quality diet category based on HEIC, while no refugees did. There were also five refugees who had poor diets, while no immigrants did. 

**Table 3:** Nutrient intake related to bone health.

Characteristics	Immigrants *n* = 33 (45.8%)	Refugees *n* = 39 (54.2%)	All participants *n* = 72 (100%)
Vitamin D intake in IU/day (Mean ± SD)	249 ± 247	181 ± 130	213 ± 195
Prevalence of vitamin D intake inadequacy [*n* (%)]	25 (81%) *	34 (97%)	59 (89%)
Calcium intake in mg	764 ± 393	676 ± 380	718 ± 386
Prevalence of calcium intake inadequacy [*n* (%)]	23 (74%)	27 (77%)	50 (76%)
Milk and alternatives			
Mean intake (servings/day)	1.9 ± 1.2	1.6 ± 1.1	1.7 ± 1.2
Meeting Canada’s Food Guide recommendations	10 (32.3%)	6 (17.1%)	16 (24.2%)
Healthy Eating Index Canada			
Mean Score	65.4 ± 7.7 *	60.4 ± 8.8	62.7 ± 8.6
Good Diet	1 (3.2%)	0 (0.0%)	1 (1.5%)
Diet Needs Improvement	30 (96.8%)	30 (85.7%)	60 (90.9%)
Poor Diet	0 (0.0%)	5 (14.3%)	5 (7.6%)

* Significantly different from refugee children, *t*-test (*P* < 0.05).

Total serum 25(OH)D (nmol/L) was significantly higher in immigrant compared to refugee children (*p* = 0.021) (Table 4). Overall, 29% of participants were vitamin D deficient and another 44% had inadequate levels of serum 25(OH)D for bone health [22]. Children spent 3.6 ± 1.6 h/day (mean ± SD) in the sun in the summer months during peak times (11:00 am–4:00 pm), which was consistent across immigration status. Very few children regularly applied sunscreen, whereas more than half of children never did (Table 4). In linear regression analyses, after controlling for possible confounders including total caloric intake, age, sunscreen use, total hours spent in the sun in the summer months during peak times, and calcium intake; dietary vitamin D intake (Standardized Regression Coefficient: 0.46 ± 0.11, *p* < 0.001), sex (Standardized Regression coefficient: −0.39 ± 0.11, *p* < 0.001), region of origin (Standardized Regression coefficient: 0.24 ± 0.11, *p* = 0.027), and length of stay in Canada (Standardized Regression Coefficient: −0.23 ± 0.11, *p* = 0.04) were found to be significant predictors of serum vitamin D status. Females—those who had been living in Canada longer, those from regions with darker skin pigmentation, and those with lower vitamin D intake—were at greater risk of low circulating vitamin D.

**Table 4:** Vitamin D status of newcomer children.

Characteristics	Immigrants *n* = 33 (45.8%)	Refugees *n* = 39 (54.2%)	All participants *n* = 72 (100%)
H/day spent in the sun during peak times (Mean ± SD)	3.6 ± 1.7	3.5 ± 1.5	3.6 ± 1.6
Sunscreen use			
always	0 (0.00%)	2 (5.1%)	2 (2.8%)
often	4 (12.1%)	0 (0.00%)	4 (5.6%)
sometimes	5 (15.2%)	10 (25.6%)	15 (20.8%)
rarely	3 (9.1%)	5 (12.8%)	8 (11.1%)
never	21 (63.6%)	22 (56.4%)	43 (59.7%)
Total serum vitamin D in nmol/L (Mean ± SD)	45.7 ± 13.9 *	37.8 ± 15.5	41.2 ± 15.2
Deficient and inadequate <50 nmol/L	19 (63.3%)	31 (79.5%)	50 (72.5%)
Sufficient ≥50 nmol/L	11 (36.7%)	8 (20.5%)	19 (27.5%)
Total body bone mineral content (TBBMC) in grams (Mean ± SD)	984.9 ± 245.0	947.8 ± 208.8	964.6 ± 224.9
Low TBBMC	13 (41.9%)	14 (35.9%)	27 (38.6%)

* Significant difference from refugees; for means a *t*-test was used and for categorical variables chi square was used.

TBBMC was low in 38.6% of participants compared to estimated values for age, sex, and ethnicity [24]. In the regression model, after controlling for possible confounders, including immigration status; food security; age; sex; region of origin; physical activity level; total caloric intake; and intakes of calcium, magnesium, phosphorus, sodium, and caffeine, height and serum vitamin D status were found to be determinants of TBBMC (Table 5). Children who were taller and had greater serum vitamin D also had greater TBBMC.

**Table 5:** Factors associated with total body bone mineral content (TBBMC) in regression analysis (using the stepwise procedure) among all subjects (*n* = 56).

**Outcome variable**	**Constant**	**Regression coefficient**				**Total *R*^2^**
		**Height (cm)**		**Serum vitamin D (nmol/L)**		
TBBMC	−1257.33	0.95 ± 0.06		0.13 ± 0.06		0.82
Partial *R*^2^		0.90		0.27		
*p*-value		<0.001		0.047		
**Outcome variable**	**Excluded variables**					
	**Sex**	**Region of origin**	**Age**	**Calcium (mg)**	**Calories (kcal)**	**Immigration Status**
TBBMC	0.01	−0.09	−0.12	0.07	0.07	0.05
Partial *R*^2^	0.03	−0.20	−0.17	0.13	0.14	0.12
*p*-value	0.832	0.147	0.227	0.353	0.323	0.408
**Outcome variable**	**Excluded variables**					
	**H/week in physical activities**	**Food security**	**Magnesium (mg)**	**Phosphorous (mg)**	**Sodium (mg)**	**Caffeine (mg)**
TBBMC	0.02	0.11	0.06	0.06	−0.04	−0.03
Partial *R*^2^	0.03	0.23	0.11	0.11	−0.08	−0.06
*p*-value	0.805	0.095	0.419	0.434	0.574	0.660

## 4. Interpretation

The HIC study is the first to evaluate vitamin D status of newcomer immigrant and refugee children aged 7–11 years in Canada. Data from a nationally representative sample showed alarmingly low 25(OH)D levels in immigrant children, particularly girls. HIC also showed a rate of 25(OH)D deficiency among newcomer immigrant and refugee children. Among these growing children, after controlling for biological and environmental co-factors, serum vitamin D was a significant predictor of TBBMC. 

Using CHMS data, we previously reported Canadian children 6–11 years had higher 25(OH)D concentrations than adolescents and adults [2]. However, non-White Canadians were less likely to achieve recommended levels. This same study showed first generation immigrant children aged 6–11 years, particularly girls, had significantly lower 25(OH)D compared to their non-immigrant counterparts. Many newcomers have darker skin pigmentation and are, therefore, at greater risk for deficiency as melanin in the skin prevents the body from synthesizing vitamin D [28]. It is likely that females were at greater risk of deficiency than males due to cultural practices associated with greater covering of females causing insufficient skin exposure to the sun even though they may have been outdoors [28]. 

In the absence of comprehensive data on dietary intakes and other health measures in CHMS, specifically in newcomer children, data from HIC provides more insight into health concerns faced by this population. Many newcomer children, particularly refugees, did not have a good quality diet including dietary sources of vitamin D. However, vitamin D intake was still the main predictor of serum vitamin D. Circulating vitamin D of 75 nmol/L is considered beneficial for multiple health outcome [29]. None of the HIC participants met the 25(OH)D level of 75 nmol/L and over 29% were vitamin D deficient. This occurred even though non-Caucasian immigrant and refugee children spent almost four hours a day outside during peak times in summer months and the majority of participants rarely or never used sunscreen. It is more difficult to get the necessary vitamin D in areas of high latitude, especially during winter months [3,4,5,6]. This may explain why those who had been in Canada longer were at greater risk of deficiency; vitamin D stores are depleted each winter and not replenished enough in the summer. Girls, children with low vitamin D intake, those from regions with darker skin, and those with longer duration of stay in Canada were at higher risk of having inadequate/deficient levels of serum vitamin D. 

Since bone mineral mass decreases with age, research has emphasized the importance of achieving optimal bone mineral accrual during childhood and adolescence, which will decrease skeletal-related health issues later in life [30,31,32,33]. Therefore, meeting requirements for vitamin D and calcium in these critical ages is crucial [34,35]. Most newcomer children in this study were not getting the recommended amounts of vitamin D and dietary calcium for bone health. Low intakes of calcium and vitamin D accord with low intakes of milk and alternatives, the main dietary sources of these two important nutrients [35]. Inadequate levels impede proper bone growth, which can result in stunting and increase the risk of developing osteoporosis in the future [4,5,22]. Serum vitamin D was a significant predictor of TBBMC in this study. TBBMC was lower than predicted values for age, sex and ethnicity in over 38% of participants. A recent Canadian study found significantly higher TBBMC values in Caucasian compared to Asian children [36]. There was no significant difference in TBBMC according to immigration status in HIC. Further research with a larger sample size is needed to examine TBBMC in children according to ethnicity. 

The relatively small sample size in the HIC study may limit the conclusions. However, the accordance of HIC findings on serum vitamin D status of immigrant children with those from the CHMS confirms the importance of HIC results. In the absence of a large-scale comprehensive study on the nutrition and health status of newcomer children, our data could provide valuable insight into this area. HIC also distinguishes refugees from immigrants. Self-reported data on dietary intake using a 24-h recall is subject to under-reporting/over-reporting and omission of frequently forgotten items. To maximize accuracy of the data, we used three 24-h recalls where our expert research personnel used proper probing and assessment aids such as food model booklets, measuring cups and spoons, and color pictures for various food items to help recall names of food items. The premise of the study is to investigate and document disparities in and between host and mobile populations that reflect biological, social and behavioral differences between these populations. Defining these differences can lead to greater awareness of their existence in health providers who may appreciate them and assist those developing programs and policies to identify and mitigate preventable outcomes. Once a framework of existing disparities is defined, further investigation to explore and delineate specific co-founding factors can be undertaken.

## 5. Conclusion

In addition to the beneficial effects of vitamin D on bone, recent data on the association between vitamin D and chronic diseases including certain types of cancers, diabetes, and multiple sclerosis, demonstrate the importance of this nutrient in growing children [34,35]. Although more research is warranted, a considerably high rate of vitamin D intake inadequacy and serum deficiency/inadequacy in newcomer children is already associated with bone mineral mass during pre-adolescence. This, therefore, requires preventive interventions to minimize the risk of serious vitamin D related diseases.
